# Focal CA3 hippocampal subfield atrophy following LGI1 VGKC-complex antibody limbic encephalitis

**DOI:** 10.1093/brain/awx070

**Published:** 2017-03-28

**Authors:** Thomas D. Miller, Trevor T.-J. Chong, Anne M. Aimola Davies, Tammy W.C. Ng, Michael R. Johnson, Sarosh R. Irani, Angela Vincent, Masud Husain, Saiju Jacob, Paul Maddison, Christopher Kennard, Penny A. Gowland, Clive R. Rosenthal

**Affiliations:** 1 Nuffield Department of Clinical Neurosciences, University of Oxford, Oxford, UK; 2 National Hospital for Neurology and Neurosurgery, London, London, UK; 3 Monash Institute of Cognitive and Clinical Neurosciences, Monash University, Clayton, Victoria 3800, Australia; 4 Department of Experimental Psychology, University of Oxford, Oxford, UK; 5 Australian National University, Research School of Psychology, Canberra, ACT, AUS; 6 Centre for Anaesthesia, Critical Care and Pain Medicine, University College London Hospital, London UK; 7 Division of Brain Sciences, Charing Cross Campus, Imperial College London, London UK; 8 Neurology Department, Queen Elizabeth Neuroscience Centre, University Hospitals of Birmingham, Birmingham, UK; 9 Neurology Department, Queen’s Medical Centre, Nottingham, UK; 10 Sir Peter Mansfield Imaging Centre, School of Physics and Astronomy, University of Nottingham, Nottingham UK

**Keywords:** limbic encephalitis, hippocampus, ultra-high resolution 7.0 T MRI, hippocampal subfields, amnesia

## Abstract

Magnetic resonance imaging has linked chronic voltage-gated potassium channel (VGKC) complex antibody-mediated limbic encephalitis with generalized hippocampal atrophy. However, autoantibodies bind to specific rodent hippocampal subfields. Here, human hippocampal subfield (subiculum, cornu ammonis 1-3, and dentate gyrus) targets of immunomodulation-treated LGI1 VGKC-complex antibody-mediated limbic encephalitis were investigated using *in vivo* ultra-high resolution (0.39 × 0.39 × 1.0 mm^3^) 7.0 T magnetic resonance imaging [*n = *18 patients, 17 patients (94%) positive for LGI1 antibody and one patient negative for LGI1/CASPR2 but positive for VGKC-complex antibodies, mean age: 64.0 ± 2.55 years, median 4 years post-limbic encephalitis onset; *n = *18 controls]. First, hippocampal subfield quantitative morphometry indicated significant volume loss confined to bilateral CA3 [F(1,34) = 16.87, P < 0.0001], despite hyperintense signal evident in 5 of 18 patients on presentation. Second, early and later intervention (<3 versus >3 months from symptom onset) were associated with CA3 atrophy. Third, whole-brain voxel-by-voxel morphometry revealed no significant grey matter loss. Fourth, CA3 subfield atrophy was associated with severe episodic but not semantic amnesia for postmorbid autobiographical events that was predicted by variability in CA3 volume. The results raise important questions about the links with histopathology, the impact of the observed focal atrophy on other CA3-mediated reconstructive and episodic mechanisms, and the role of potential antibody-mediated pathogenicity as part of the pathophysiology cascade in humans.

## Teaser

Autoantibodies linked with voltage-gated potassium channel (VGKC)-complex antibody-mediated limbic encephalitis (LE) bind to hippocampal subfields. Miller *et al*. report that chronic LGI1 antibody VGKC-complex LE is associated with focal CA3 atrophy on 7.0-Tesla neuroimaging and autobiographical episodic amnesia. The results point to antibody-mediated pathogenicity and CA3-mediated impairment of episodic memory.

## Introduction

Autoantibodies to two main antigenic components of voltage-gated potassium channel (VGKC)-complex—LGI1 and CASPR2 proteins—are preferentially expressed in two rodent hippocampal subfields, cornu ammonis (CA)3 and CA1 (Irani *et al.*, 2010). Encephalitis in patients positive for VGKC-complex, LGI1, or CASPR2 antibodies can present with inflammatory changes of the medial temporal lobe on acute MRI (Kotsenas *et al.*, 2014), and whole-hippocampal atrophy in the convalescent and chronic phases (Irani *et al.*, 2013; Malter *et al.*, 2014). Neuropsychological assessment, following immunomodulation therapy, reveals substantial variability in outcome (Chan *et al.*, 2007; Frisch *et al.*, 2013; Bettcher *et al.*, 2014; Butler *et al.*, 2014; Malter *et al.*, 2014), unlike the more severe deficits often observed in progressive medial temporal lobe diseases such as Alzheimer’s disease (Wolk *et al.*, 2011).

Evidence of *in vivo* pathology restricted to one or two human hippocampal subfields is uncommon. Focal lesions in CA1 have been associated with transient epileptic amnesia (Bartsch *et al.*, 2011), whereas in a recent case study involving hypoxic-ischaemic brain injury, the lesion profile was confined to the bilateral dentate gyrus and a portion of CA3 (Baker *et al.*, 2016). In both cases, the subfield lesions were associated with episodic amnesia (Bartsch *et al.*, 2011; Baker *et al.*, 2016). To our knowledge, pathology confined to human CA3 has not been previously reported *in vivo*, but evidence from molecular imaging in a rodent model and functional MRI in humans indicates CA3 activity is associated with episodic retrieval (Bonnici *et al.*, 2013; Lux *et al.*, 2016).

In the current study, the human hippocampal subfield targets of immunomodulation-treated chronic LGI1 VGKC-complex antibody-mediated limbic encephalitis were investigated for the first time using ultra-high spatial resolution MRI conducted at 7.0 T (17/18 patients were positive for LGI1 antibody and one patient was VGKC-complex-antibody positive and negative for LGI1 and CASPR2 antibodies). Whole-hippocampal images (390 µm^2^ in-plane spatial resolution) were acquired to conduct detailed 3D *in vivo* hippocampal subfield quantitative manual morphometry of five subfields (CA1–3, dentate gyrus, subiculum). By comparison, previous reports have conducted automated volumetry of mesiotemporal structures, based on whole-brain images acquired at ≤3.0 T and lower spatial resolution ≥1.0 mm^3^ (Irani *et al.*, 2013; Finke *et al.*, 2017), and either collapse across individual subfields or report total hippocampal volume. In addition, whole-brain, voxel-by-voxel morphometry was conducted on high-resolution (600 µm isotropic) T_1_-weighted images to test for grey matter reductions across the rest of the brain. Finally, to the extent that CA3 interacts with CA1 and the dentate gyrus to support episodic memory (Baker *et al.*, 2016), we tested the hypothesis that CA3 and/or CA1 atrophy would be linked with selective deficits in the retrieval of experiential, first person components of episodic but not semantic memory detail for the same event.

## Materials and methods

### Participants

Eighteen patients (mean age: 64.0 ± 2.55 years; range: 24–71; 15 male), all with a formal clinical diagnosis of VGKC-complex antibody-mediated limbic encephalitis, participated [mean titre = 2270 pmol/l; interquartile range = 648–3922; standard deviation (SD) = 1836]. Clinical records were retrospectively interrogated for time-to-treatment from first symptom, first-line treatments (Supplementary material), and evidence of medial temporal lobe involvement on presentation. Seventeen of the patients were LGI1 positive and one was positive for VGKC-complex antibodies and negative for LGI1 and CASPR2. Patients were recruited if they were >12-months post-symptom onset (median time from first symptom-to-treatment = 3 months) and considered clinically stable. Eighteen healthy age-matched controls were recruited (64.6±1.94 years; range: 22–76; 11 male), and had no history of cognitive, psychiatric, or neurological illness. The local research ethics committee approved the protocol. Informed written consent was obtained from all participants for all procedures.

### MRI

#### Image acquisition

All participants were scanned to acquire three MRI sequences on a 7.0 T whole body MRI scanner (Achieva, Philips Healthcare): (i) a rapid whole-head sagittal T_1_-weighted localizer image to verify head position; (ii) a T_2_-weighted 3D fast spin-echo coronal sequence with the refocusing pulse adjusted to optimize contrast covering both hippocampi (voxel size, 0.39×0.39×1.0 mm^3^); and (iii) a 3D whole-brain T_1_-weighted phase sensitive inversion recovery sequence providing inherent receiver bias field correction in the image reconstruction and using a tailored radiofrequency pulse for magnetization inversion at ultrahigh field (isotropic voxel size, 0.6 mm^3^). Scan parameters are reported in the Supplementary material.

#### Hippocampal subfield segmentation

**Figure 1:**
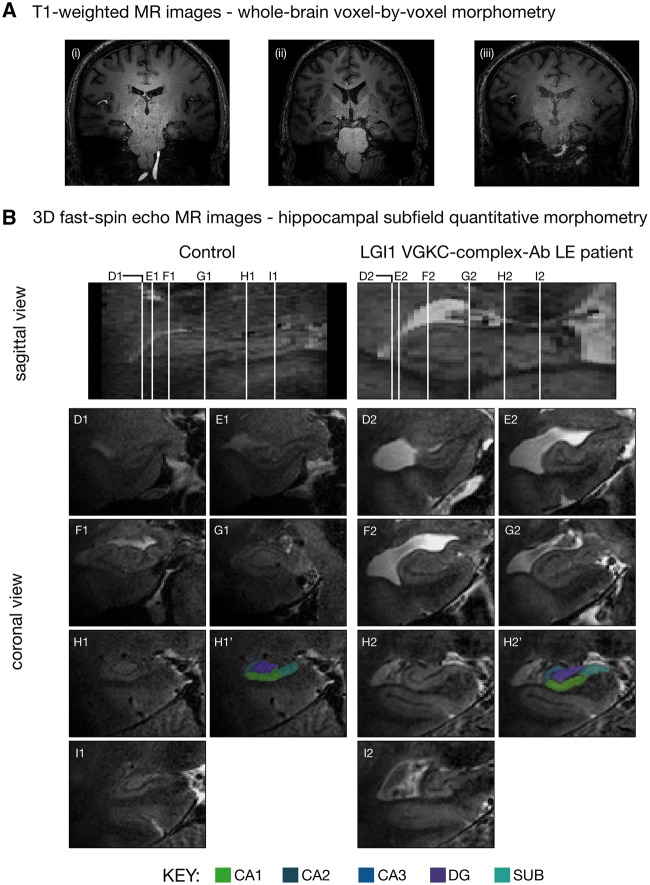
**7.0 T field strength T_1_-weighted whole-brain and 3D fast-spin echo whole-hippocampal MRIs obtained from LGI1 VGKC-complex antibody-mediated limbic encephalitis (VGKC-complex-Ab LE) group and age-matched control group.** (**A**) T_1_-weighted (0.6 × 0.6 × 0.6 mm^3 ^resolution) coronal images from three patients in the LGI1 VGKC-complex antibody-mediated limbic encephalitis group (**i–iii**) illustrating significant *in vivo* hippocampal atrophy. Normalized grey matter from controls and the patients with hippocampal damage derived from whole-brain voxel-based morphometry analysis were contrasted using a two sample *t*-test and thresholded at *P < *0.05 family-wise error corrected for multiple comparisons with SPM12. This whole-brain analysis revealed no significant differences between the grey matter volumes of patients and controls (see ‘Results’ section and Supplementary material). (**B**) Native coronal images from whole-hippocampal ultra-high resolution (0.39 × 0.39 × 1.0 mm^3^ resolution) 3D fast-spin echo sequence. Manual hippocampal subfield (subiculum, CA1–3, dentate gyrus) segmentation was conducted along the full longitudinal axis and revealed selective lesions to bilateral CA3. Examples from applying the manual hippocampal subfield segmentation protocol at 7.0 T in a control (**1**) and in a patient with LGI1 VGKC-complex antibody-mediated limbic encephalitis (**2**). Each of the white lines (**D–I**) on the sagittal view of the hippocampus corresponds to six example coronal locations along the anterior–posterior axis. **H1’** and **H2’** illustrate the segmentation protocol used in this study. The colour key corresponding to the SUB (subiculum), CA1, CA2, CA3, DG (dentate gyrus) hippocampal subfields is shown below the image.

Quantitative morphometry of CA1–3, dentate gyrus, and the subiculum was conducted on left and right hippocampal subfields, deriving 10 volumes (mm^3^), and guided by a previously described 7.0 T manual segmentation protocol (Fig. 1) (Wisse *et al.*, 2012), modified so that CA2 and the boundary between CA1 and the subiculum were additionally delineated on each slice. Manual segmentation was performed on the 3D fast-spin echo images using the freehand spline drawing and manual segmentation tools in ITK-SNAP 3.2 (http://www.itksnap.org). To evaluate intra-rater reliability, all hippocampi were fully re-segmented after a month had elapsed. A subset of patient and control scans also underwent manual segmentation by a second rater to generate inter-rater reliability indices. Both raters were blinded to the identity of all scans.

#### Whole-brain voxel-by-voxel morphometry

An automated voxel-based morphometry analysis, implemented within SPM12 software and executed in Matlab R2013a, was conducted on the T_1_-weighted whole-brain images to test for grey matter differences between the patients and controls (Supplementary material). The T_1_-weighted images were also used to derive total intracranial volumes to compensate for interindividual variability in head size.

### Behaviour

#### Neuropsychological assessment and postmorbid autobiographical memory

To obtain a detailed neuropsychological profile, the patients underwent a comprehensive assessment using standardized neuropsychological tests. Separately, the ability of patients (*n = *16) and controls (*n = *16) to recollect postmorbid experiential, episodic, and semantic event details was also assessed on an objective, parametric, text-based procedure—the autobiographical interview (Levine *et al.*, 2002)—that has been administered to study autobiographical memory in health and in disease (Addis *et al.*, 2007). See Supplementary material for further details.

### Statistical analyses

Intra- and inter-rater reliability of manual segmentation was assessed using the Dice similarity indices (DSIs) of geometric overlap for the five subfields and derived using Convert3D (www.itksnap.org). In addition, intra-class correlation coefficients (ICCs) were used to calculate the agreement between twice-repeated manual segmentations of all five subfields as a function of side (left, right) and twice-repeated scoring of data acquired on the autobiographical interview. A two-way mixed-model design was used to test the degree of absolute agreement.

Hippocampal subfield volumes were examined using a mixed-model omnibus ANOVA and planned between-group comparisons. Contrasts involving unequal subgroups were conducted by means of non-parametric median test and Kruskal-Wallis test and *post hoc* tests. Contrasts following from these parametric and non-parametric tests were assessed with the alpha criterion corrected for multiple comparisons using the Bonferroni-Holm procedure. Volumetric data were assessed against other variables with robust multiple linear regression conducted using Huber’s method of correction for outliers. Statistics were computed using IBM SPSS Version 24.0 (IBM Corp.) and NCSS.

## Results

### MRI

#### Hippocampal subfield segmentation

The ICCs for each subfield indicate a high-to-excellent consistency for all hippocampal subfields in patients (median: 0.965) and controls (median: 0.961). Intra-rater DSIs for each subfield revealed high-reliability for patients and controls (median patient and control DSIs generalized across all subfields: 0.76 and 0.79, respectively). Inter-rater DSIs of two independent raters for patients and controls were also reliable (median patient and control DSIs generalized across all subfields: 0.75 and 0.74, respectively).

#### Focal bilateral CA3 atrophy

Mean subfield volumes, corrected for differences in total intracranial volume, are reported in Table 1. A three-way mixed-model ANOVA was conducted on the subfield volumes, with two within-subjects variables (subfield and side) and one between-subjects variable (group). Mauchly’s test demonstrated that the assumption of sphericity had been violated for side [χ^2^(9) = 81.61, *P* < 0.001] and subfield by side [χ^2^(9) = 82.44, *P < *0.001], therefore, degrees of freedom were corrected using Greenhouse-Geisser estimates (ɛ = 0.46 and 0.49, respectively). There were significant main effects of group [*F*(1,34) = 6.08, *P* = 0.019], side [*F*(1,34) = 44.81, *P < *0.0001], and subfield [*F*(1.84,62.49) = 346.80, *P < *0.0001]. Significant two-way interactions were observed between group and subfield [*F*(1.84) = 4.21, *P* = 0.022] and between side and subfield [*F*(1.96,66.75) = 20.19, *P < *0.0001]. The three-way interaction between group, subfield, and side was not significant [*F*(1.96) = 1.89, *P* = 0.16].

**Table 1:** Hippocampal subfield volumes in the LGI1 VGKC-complex antibody-mediated limbic encephalitis patient group (*n* = 17 LGI1 positive and one patient who was LG1I and CASPR2 negative but VGKC-complex antibody-positive) and control (*n* = 18) group

Subfield	Mean total subfield volumes		
	LGI1 VGKC-complex antibody-mediated limbic encephalitis group	Control group	Planned comparisons
CA1	986 (±73, 311)	1171 (±38, 162)	ns
CA2	171 (±9, 40)	180 (±10, 41)	ns
CA3[Table-fn tblfn1]	377 (±19, 82)	525 (±30,128)	−28%
Dentate gyrus	615 (±42,180)	659 (±23,95)	ns
Subiculum	520 (±33,140)	603 (±22,95)	ns

Values are mean, mm^3^, (±SEM, SD).

Volumes were normalized to the total intracranial volumes obtained from the voxel-based morphometry analyses. Volumes are collapsed across the left and right hippocampi because there was no significant interaction term between group, side (left, right), and subfield.

*Significant at the alpha criterion based on Bonferroni-Holm correction for multiple comparisons, following mixed-model ANOVA; significant mean percentage reduction shown for CA3; ns = non-significant at alpha criterion corrected for multiple comparisons. Total intracranial volume was derived by applying the sequence of unified segmentation, as implemented in SPM12, to the whole-brain T_1_-weighted 7.0 T images that were also acquired from each participant.

**Figure 2:**
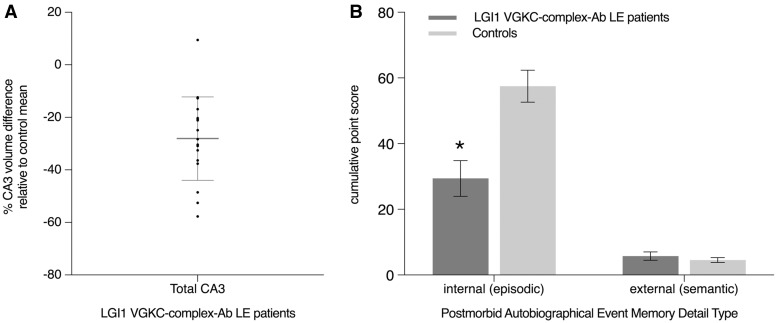
**CA3 atrophy and impairment of autobiographical episodic memory in the LGI1 VGKC-complex antibody-mediated limbic encephalitis group**. (**A**) Results from hippocampal subfield segmentation in the LGI1 VGKC-complex antibody-mediated limbic encephalitis patient group. Total intracranial volume-normalized total CA3 subfield volume reductions relative to the control group mean. Significant CA3 atrophy was seen in the LGI1 VGKC-complex antibody-mediated limbic encephalitis group relative to age-matched control group [mean reductio*n = *28%; *F*(1,34) = 16.87, *P* < 0.0001; Cohen’s *d* = 1.37]. Hippocampal subfield segmentation was based on 3D fast-spin echo images acquired at 390 μm^2^ in-plane spatial resolution. Mean total intracranial volume-normalized total CA3 subfield volumes are reported in Table 1. The error bar corresponds to the standard deviation; and (**B**) behavioural measure of postmorbid autobiographical episodic and semantic memory. Cumulative (summed across the general and specific probes) mean number of internal (episodic) and external (non-episodic, mainly personal semantic) details generated on the autobiographical interview, as a function of group. Recollected events correspond to memories formed postmorbidly. Patients exhibited a significant deficit in the recollection of episodic details relative to controls *[*F*(1,30) = 14.94, *P* = 0.001, Cohen’s *d* = 1.37], whereas the recollection of semantic details from the same autobiographical event in the patients was comparable to the control group [*F*(1,30) = 0.71, *P* = 0.41, Cohen’s *d* = 0.30]. Error bars correspond to the SEM.

Planned comparisons revealed a significant reduction in total CA3 volume–collapsed across left and right CA3 due to the absence of a significant three-way interaction–in the patients relative to controls [*F*(1,34) = 16.87, *P* < 0.0001; mean reduction* = *28%, Cohen’s *d* = 1.37] (Fig. 2A), whereas the differences in subiculum, CA1, CA2, and dentate gyrus volumes were not statistically significant at the alpha corrected for multiple comparisons (Cohen’s *d* all < 0.8). Importantly, the pattern of results were unchanged when these data were reanalysed with 17/18 patients who were LGI1 antibody-positive (Supplementary material).

Retrospective assessment of neuroradiological reports on the clinical MRI scans acquired at presentation revealed medial temporal lobe MRI signal hyperintensities in five of the 18 patients. Even though hyperintense medial temporal lobe signal progresses to hippocampal atrophy as the signal declines (Chan *et al.*, 2007; Bien *et al.*, 2012), hyperintensity on presentation was not a necessary condition for chronic hippocampal subfield atrophy and did not affect the CA3 atrophy (Supplementary material). In addition, antibody titre at presentation, listed in Supplementary Table 1, did not predict the magnitude of CA3 atrophy (*β*_1_ = 0.002, *R^2^* = 0.07, *t* = 1.07, *P* = 0.30). Likewise, CA3 atrophy was not predicted by median disease duration at 7.0 T MRI assessment (*β*_1_ = 1.18, *R*^2 ^= 0.04, *t* = 0.84, *P* = 0.42; median* = *4 years; range: 1–9 years).

#### Early and later intervention were both associated with CA3 subfield atrophy

Patients were dichotomized into early and later intervention groups with 3-month cut-off (Finke *et al.*, 2012) (see Supplementary material for first-line treatment). An independent-samples median test between patients receiving early intervention (i.e.<3 months from symptom-onset), and later intervention (i.e. >3 months), and controls revealed a significant between-group difference in CA3 subfield volume [χ^2^(2) = 8.13, *P* = 0.017]. Pairwise comparisons revealed that both intervention groups were associated with significant CA3 volume loss compared to controls (early: *z* = 4.89, *P* = 0.027, Cohen’s *d* = 1.21; later: *z* = 8.13, *P* = 0.004, Cohen’s *d* = 1.40).

#### Group voxel-by-voxel based morphometry

A voxel-by-voxel contrast of normalized grey matter conducted using a two-sample *t*-test thresholded at *P < *0.05 family-wise error corrected for multiple comparisons across the whole-brain revealed no suprathreshold clusters of grey matter volume loss in the patients relative to controls, and no significant suprathreshold clusters for the reverse between-group contrast (LGI1 VGKC-complex antibody-mediated limbic encephalitis > controls). This result aligns with other recent studies that have conducted comparable voxel-based morphometry analyses (Wagner *et al.*, 2015; Finke *et al.*, 2017).

### Behaviour

#### Postmorbid autobiographical memory and neuropsychological profile

Results from the autobiographical interview revealed significant main effects of group (patients, controls) [*F*(1,30) = 11.59, *P* = 0.002] and memory detail type (internal, external) [*F*(1,30) = 123.24, *P < *0.0001], and a significant interaction effect [*F*(1,30) = 18.07, *P < *0.0001]. Planned comparisons indicated a selective and severe (mean reduction* = *48.9%) loss of internal (episodic) [*F*(1,30) = 14.94, *P = *0.001, Cohen’s *d* = 1.37] but no loss of external (semantic) [*F*(1,30) = 0.71, *P* = 0.41, Cohen’s *d* = 0.30] autobiographical memory detail in the patients compared to the controls (Fig. 2B and Supplementary material). By contrast, the patients were unimpaired on the indices of intelligence, executive function, attention, language, visuomotor, and visuoconstructive skills (Table 2), and thus the loss in autobiographical episodic memory is unlikely to be secondary to deficits in cognitive faculties that are strongly linked with memory performance such as attention or executive function.

**Table 2:** Neuropsychological domain performance of the patients in the LGI1 VGKC-complex-Ab LE patient group (*n* = 17 LGI1 antibody positive and one patient who was LG1I and CASPR2 negative but VGKC-complex antibody positive)

Neuropsychological	*n*	Average *z*-score	SEM	*t*	*d.f.*	*P*-value
Intelligence	16	0.90	0.20	4.46	15	<0.001
Verbal memory	17	−0.34	0.20	−1.70	16	ns
Visual memory	17	−0.17	0.16	−1.06	16	ns
Recognition memory	17	−0.07	0.19	−0.37	16	ns
Attention	15	0.22	0.14	1.62	14	ns
Executive function	17	0.65	0.16	4.13	16	0.001
Language	17	0.51	0.22	2.35	16	0.032
Visuomotor skills	17	0.17	0.10	1.80	16	ns
Visuoconstructive skills	17	−0.01	0.32	−0.03	16	ns

Domain scores are based on neuropsychological subtests that are described in the Supplementary material. Notably, delayed verbal recall performance (which contributed to the verbal memory domain) was significantly different from normative data [*n* = 17, average *z*-score = −0.68, SEM = 0.24, *t*(16)= − 2.83, *P* = 0.012].

*Patient group statistically different from normative data, P < 0.05, two-tailed; n = number of patients tested. Delayed verbal recall was comprised of Logical Memory II, Logical Memory II themes and Word Lists II (WMS-III) and People Recall Test.

Notably, the variability in total CA3 volume of patients and controls, i.e. with CA3 volume as a continuous independent variable, selectively predicted the amount of internal (episodic) detail retrieved [*t* = 2.43, *P* = 0.021, *R*^2 ^= 0.17, *β*_1_ = 0.07], but not the external (semantic) detail score [*t* = 0.92, *P* = 0.363, *R*^2 ^= 0.03, *β*_1_ = 0.004].

## Discussion

Ultra-high resolution anatomical magnetic resonance neuroimaging conducted at 7.0 T and quantitative hippocampal subfield morphometry in chronic LGI1 VGKC-complex antibody-mediated limbic encephalitis revealed a restricted subfield lesion profile limited to the CA3. The results redefine the *in vivo* characterization of chronic LGI1 VGKC-complex antibody-mediated limbic encephalitis in four areas: (i) hippocampal atrophy was selective, affecting bilateral CA3, and occurred in the absence of significant grey matter loss in brain regions outside of the hippocampus; (ii) CA3 atrophy was evident following early and later intervention; (iii) CA3 atrophy was evident in patients with and without hyperintense signal on presentation; and (iv) focal CA3 atrophy was associated with severe impairment of postmorbid episodic but not semantic autobiographical memory. The patients were otherwise intact on the indices of verbal, visual, and recognition memory. The dissociation between these indices based on standardized tests of memory and the measure of autobiographical episodic memory is consistent with the hypothesis that retrieving autobiographical event knowledge is qualitatively different from other forms of episodic retrieval (Roediger and McDermott, 2013).

The results demonstrate the utility of conducting MRI studies of medial temporal lobe pathology at submillimetre spatial resolution. First, it enabled the detection of specific subfield pathology, and, second, it allowed comparison of neuroradiological evidence in humans with autoantibody binding patterns in rodents, which have been described as part of the pathophysiological cascade that involves VGKC-complex expression in the hippocampus (Vincent *et al.*, 2011). Unlike CA3, the between-group CA1 contrast did not reach statistical significance when corrected for multiple comparisons. The selectivity aligns with evidence of greater neuronal loss in CA3 than CA1 following seizures in homozygous LGI1 knockout mice (Chabrol *et al.*, 2010). CA3 volume loss may have arisen due to the particular vulnerability of CA3 versus CA1 to excitotoxic lesions associated with seizures, given that IgG containing LGI1 antibodies induce population epileptiform discharges in CA3 pyramidal neurons *in vitro* (Lalic *et al.*, 2011), or from complement-mediated fixation of bound antibodies (Bien *et al.*, 2012).

Potential pathomechanisms related to human LGI1 antibodies from rodent models involve interference with the LGI1–ADAM22 interaction and reduced postsynaptic AMPARs *in vitro* (Ohkawa *et al.*, 2013). Furthermore, even though anatomical localization of *LGI1* gene transcripts indicates enrichment in CA3 of the adult human brain (Hawrylycz *et al.*, 2012), which aligns with the expression of *LGI1* gene transcripts in mouse dentate gyrus and CA3 (Herranz-Perez *et al.*, 2010), the pathogenic role and antigenic targets of LGI1 autoantibodies need to be determined in humans. To date, evaluations have centred on associations between antibody levels and clinical features, the detection of these antibodies in CSF as well as serum, and the use of *in vivo* and *in vitro* experimental animal models to develop similar changes to those seen in the human disease. Future studies will need to contrast evidence from longitudinal *in vivo* ultra-high resolution MRI with results from *ex vivo* MRI acquisition to bridge the gap with nascent immunohistological data that have identified CA4/dentate gyrus with volume loss (Bauer and Bien, 2016).

Patients in the early and later intervention subgroups both received high-dose corticosteroids as first-line treatment. At present, there is no standard treatment for autoimmune encephalitis, but rapid immunotherapy with high-dose corticosteroids, intravenous immunoglobulin or plasma exchange remain equivocal first-line therapies (Irani *et al.*, 2013). Larger and preferably comparable numbers of patients in each subgroup will need to be investigated to assess more fully the impact of early intervention. Furthermore, the null effect of the timing of treatment intervention on CA3 atrophy and prior evidence that hyperintense medial temporal lobe signal progresses to hippocampal atrophy as the signal declines may reflect the involvement of other mechanisms, such as an initial, T cell-mediated inflammatory responses that enable specific antibodies to enter the CNS and initiate irreversible tissue damage in CA3.

The link between focal CA3 atrophy and impaired autographical episodic memory elaborates on the chronic behavioural phenotype of VGKC-complex antibody-mediated limbic encephalitis to include amnestic pathology beyond reports of impaired verbal and figural memory (Frisch *et al.*, 2013; Bettcher *et al.*, 2014; Butler *et al.*, 2014; Malter *et al.*, 2014). Critically, prior reports did not focus on specific mnemonic operations that are reliant upon CA3 integrity. CA3 is an area involved in episodic-like recall and mental imagery (Bonnici *et al.*, 2013). CA3 is also associated with the capacity to reconstruct and recall distinctive, precise details of newly acquired episodic memories (Chadwick *et al.*, 2014), and these operations can be assessed with extended assays of autobiographical episodic memory such as the autobiographical interview (Levine *et al.*, 2002; Addis *et al.*, 2007). The loss of internal detail on the autobiographical interview likely reflected a loss of such CA3-mediated features of episodic memory, and is compatible with evidence that focal lesions involving human CA1 or the dentate gyrus (Bartsch *et al.*, 2011; Baker *et al.*, 2016), the other two subfields of hippocampal trisynaptic circuit that subtends episodic memory, are sufficient to induce severe episodic amnesia. Future studies will need to address the impact of the observed focal atrophy on other CA3-mediated reconstructive, computational, and episodic mechanisms alongside work identifying the specific pathomechanisms in humans, because these data will be key to developing optimized approaches to prevention, detection, intervention, and clinical management.

## Funding

The work was supported by the Medical Research Council (UK) and Engineering and Physical Sciences Research Council (P.A.G.), National Institute for Health Research (T.W.C.N.), National Institute for Health Research (NIHR) Oxford Biomedical Research Centre based at Oxford University Hospitals NHS Trust and University of Oxford (C.R.R., A.MA.D., C.K., & A.V.), John Fell OUP Fund (C.R.R, C.K.), Clinical Training Fellowship from the Guarantors of Brain (T.D.M.), the Patrick Berthoud Charitable Trust (T.D.M), the Encephalitis Society (T.D.M), and the Wellcome Trust (M.H.).

## Conflicts of interest

Professor Angela Vincent and the Department of Clinical Neurology in Oxford receive royalties and payments for antibody assays and Angela Vincent is the named inventor on patent application WO/2010/046716 entitled ‘Neurological Autoimmune Disorders’. The patent has been licensed to Euroimmun AG for the development of assays for LGI1 and other VGKC-complex antibodies. Sarosh R. Irani is one of the co-inventors and also receives a proportion of royalties. Dr Irani also reports personal fees from MedImmune, grants from Wellcome Trust, grants from UCB Pharma, grants from BMA, Fulbright UK, US Commission, and the MS Society, Department of Health UK, Vera Down grant, and Epilepsy Research UK, outside the submitted work. Dr Irani served on the scientific advisory board for Encephalitis Society and MedImmune; received honoraria from Movement Disorder Society and Bethel Epilepsy Symposium. Dr Jacob has been a scientific advisory board member for Alexion and Alynylam pharmaceuticals. None of the other authors have a conflict of interest to declare.

## Supplementary material

Supplementary material is available at *Brain* online.

## Supplementary Material

Supplementary Data

## Abbreviations

CA: cornu ammonis
VGKC: voltage-gated potassium channel
