# Supplementary material for: Focal CA3 hippocampal subfield atrophy following LGI1 VGKC-complex antibody limbic encephalitis
Source: Brain. 2017 Mar 28;140(5):1212–9. doi: 10.1093/brain/awx070 (PMC5405234; doi:10.1093/brain/awx070)
Supplement: Supplementary Data [file awx070_Supp.pdf]

Supplementary Material

Methods

Participants

First-line treatment in early and later intervention subgroups

First-line treatment for the early intervention group (<3 months) was high-dose oral prednisolone for three patients and one patient also had contemporaneous intravenous immunoglobulin (mean age=58.4 years, SD=10.5). In the later intervention group (mean age=63.8 years, SD=13.07), five patients received high-dose oral prednisolone, three patients received pulsed high-dose methylprednisolone, two patients had combined high-dose corticosteroid therapy with methylprednisolone and oral prednisolone, one patient received intravenous immunoglobulin monotherapy, one patient received high-dose oral prednisolone and intravenous immunoglobulin, and two patients did not receive any intervention.

MRI

Neuroimaging and Segmentation Protocols

All participants were scanned to acquire three MRI sequences on a 7.0-Tesla whole body magnetic resonance imaging scanner (Achieva, Philips Healthcare), operated with a volume-transmit 32-element receive coil array: (1) a rapid whole-head sagittal T1-weighted localizer image to verify head position and plan an oblique coronal volume-of-interest oriented perpendicular to the anterior-posterior axis of the hippocampus; (2) a three-dimensional T2-weighted fast spin-echo sequence with the refocusing pulse adjusted to optimize contrast over both hippocampi, with the following parameters: Repetition Time (TR) = 2500 ms; Echo Time (TE) = 397 ms; TSE factor = 60; flip angle = 90°; voxel size, 0.39 × 0.39 × 1.0 mm^3; field-of-view, (FH)220 × (RL)170.58 mm^2; and, 52 contiguous coronal sections, acquired perpendicular to the hippocampal axis, covering the full longitudinal axis of both hippocampi; and, (3) a three-dimensional whole-brain T1-weighted Phase Sensitive Inversion Recovery sequence (Mougin *et al.*, 2015) providing inherent receiver bias field correction in the image reconstruction and using a Tailored RF Pulse for Magnetization Inversion at Ultrahigh Field (Hurley *et al.*, 2010), with the

following parameters: 200 transverse slices, Repetition Time (TR) = 12 ms, Echo Time (TE) = 5.8 ms, flip angle = 8°, voxel size = 0.6 × 0.6 × 0.6 mm^3, field-of-view = 200(AP) × 181(RL) × 120(FH) mm^3.

The T1-weighted sequence was used as the basis for the VBM analyses and to derive total intracranial volumes to compensate for inter-individual variability in head size (Malone *et al.*, 2015). The coronal and sagittal images in Figure 1 were obtained by loading the data from the three-dimensional T2-weighted fast spin-echo sequence and three-dimensional whole-brain T1-weighted Phase Sensitive Inversion Recovery sequence into ITK-SNAP 3.2 (Yushkevich *et al.*, 2006) (http://www.itksnap.org). As noted in the main text, ITK-SNAP 3.2 was also used to conduct hippocampal subfield volumetric morphometry.

The optimised 3D-FSE sequence was obtained in addition to the whole-brain T1-weighted image because VBM automated segmentation of whole-brain images can fail to detect restricted hippocampal atrophy (Keller and Roberts, 2008; Wagner *et al.*, 2015) and is not designed to report grey matter volume loss described at the level of individual hippocampal subfields. Furthermore, automated hippocampal subfield segmentation (1) needs to be validated when applied to clinical populations (de Flores *et al.*, 2015) and/or on images at ≥3.0-Tesla signal strength to address particular issues in ultra-high field MRI such field inhomogeneity; (2) may collapse across subfield boundaries such as CA2 and CA3 that can be separated with manual quantitative morphometry; and, (3) may incorporate geometric as well as anatomical rules (Wisse *et al.*, 2014a). Improvements, however, continue apace with these automated schemes and will enable future work to compare manual segmentation with automated hippocampal subfield segmentation (de Flores *et al.*, 2015).

Whole-brain voxel-by-voxel morphometry

T1-weighted images from all participants in the LGI1 VGKC-complex-Ab LE group and age-matched control group were bias-corrected and the brain segmented into grey and white matter and CSF probability maps using the unified segmentation approach (Ashburner and Friston, 2005). Inter-subject iterative registration of the grey and white matter segments was performed using the Dartel toolbox (Ashburner, 2007). SPM12 failed to register one patient's scan and so the scan was removed from further VBM analyses. The resulting Diffeomorphic Anatomical Registration Through Exponentiated Lie algebra template and deformations were used to normalize grey and white matter probability maps to the stereotactic space defined by the

Montreal Neurological Institute template. The normalization procedure involved modulating the grey matter probability maps by the Jacobian determinants of the deformation field and smoothing with an isotropic Gaussian kernel (8-mm, full-width at half-maximum). Voxels with grey matter values <0.2 (absolute threshold masking) were excluded to avoid edge effects between the tissue types. Total intracranial volumes were included into the model as a covariate of no interest.

The VBM analysis included, for example, voxels in areas adjacent to the hippocampus and other sites enriched in LGI1, such as those reported in a study examining the anatomical localisation of gene transcripts of the LGI1 family (Herranz-Perez *et al.*, 2010). Our VBM analysis was also able to detect, if present, grey matter loss in subcortical regions such as the basal ganglia, insula cortex, hypothalamus or parahippocampal gyrus, which are other less common regions associated with high signal change on T2-weighted scans acquired in encephalitis patients positive for LGI1 antibodies.

Behaviour

Neuropsychology

Neuropsychological assessment was conducted in the LGI1 VGKC-complex-AB LE patient group on the following subtests: *General intelligence:* Wechsler Adult Intelligence Scale (WASI) – Similarities and Matrix Reasoning (Weschler, 1997); *Verbal memory:* Logical Memory I and II, Logical Memory I and II themes, and Word Lists I and II from Wechsler Memory Scale-III (WMS-III; (Wechsler, 1997) and Doors and People – People and People Recall Tests (Baddeley *et al.*, 1994); *Visual memory:* Rey-Osterrieth complex figure immediate-recall (Osterrieth, 1944) and Doors and People – Shapes Test and Visual forgetting scores (Baddeley *et al.*, 1994); *Recognition memory:* Words Lists II recognition (WMS-III; (Wechsler, 1997)), Recognition Memory Test for Words and Faces (Warrington, 1984) and Doors and People – Names and Doors Tests (Baddeley *et al.*, 1994); *Attention:* All subtests of the Test of Everyday Attention (Robertson *et al.*, 1994); *Language:* Graded Naming Test, Letter Fluency from the Verbal Fluency Test and Category Fluency from the Verbal Fluency Test (Delis-Kaplan Executive Function System, D-KEFS; (Delis *et al.*, 2001)) and the Camel and Cactus Test (Bozeat *et al.*, 2000); *Executive function:* Category Switching from the Verbal Fluency Test, Number-Letter Switching from the Trail Making Test and Colour-Word Interference Test (D-KEFS; (Delis *et al.*, 2001)), and Digit Span (WMS-III)

(Wechsler, 1997)); *Visuoconstruction:* Rey complex figure copy (Osterrieth, 1944); and, *Visuomotor:* Visual Scanning, Number Sequencing, Letter Sequencing, and Motor Speed (all from the Trail Making Test; D-KEFS; (Delis *et al.*, 2001)). Scores on the standardised neuropsychological tests were first transformed into age-corrected standard values, where available, and then transformed into *z*-scores and averaged to derive composite scores corresponding to indices for the respective cognitive domains.

No significant deficits were evident outside of delayed verbal recall – comprised of Logical Memory II, Logical Memory II themes and Word Lists II (WMS-III) and People Recall Test – on the range of neuropsychological tests (see Table 2), and are broadly in line with the neuropsychological profile previously reported in six of the current 18 patients (McCormick *et al.*, 2016, 2017). Furthermore, the severe deficits in episodic recollection evident on the autobiographical interview is not merely due to a loss of executive function or attention because the corresponding indices did not reveal deficits, which is in line with results that suggest the reconstitution of attentional-executive function following treatment (Frisch *et al.*, 2013). The results also demonstrate the merit of testing episodic memory with both standardized tests and with measures that provide extended, objective quantitative assessments of autobiographical episodic memory, because nascent evidence indicates there is little neurocognitive or behavioural overlap (McDermott *et al.*, 2009; Palombo *et al.*, 2015). The results are also compatible with the proposal that the retrieval of autobiographical event knowledge is qualitatively different from other forms of episodic retrieval (Roediger and McDermott, 2013).

Postmorbid autobiographical episodic and semantic memory

Anterograde (postmorbid) first-person autobiographical episodic memory was assessed because it is widely regarded to be dependent on an intact hippocampus in both human (Squire and Alvarez, 1995; Nadel and Moscovitch, 1997; Bontempi *et al.*, 1999; Frankland and Bontempi, 2005) and experimental (Lux *et al.*, 2016) animal lesion studies.

Anterograde autobiographical memory was investigated under the standard retrieval conditions of autobiographical interview (Levine *et al.*, 2002). The autobiographical interview has been used previously to study autobiographical episodic memory in both health and disease (Addis *et al.*, 2007; Rosenbaum *et al.*, 2008), and is designed to separate internal details (episodic recollection - the re-experiencing of all aspects of an event) from external details (non-episodic;

e.g., semantic memory) related to autobiographical information (Levine *et al.*, 2002). The basic method applied when scoring the autobiographical interview aligns with standardized tests that examine the retrieval of narrative-based details. In addition, in the autobiographical interview, the details are categorized according to whether they reference a unique episodic aspect of the event in order to distinguish these from generic or semantic content of the memory (Levine et al., 2002).

Data on the autobiographical interview were obtained from 16 of the 18 LGI1 VGKC-complex-Ab LE patients and 16 participants in the age-matched 7.0-Tesla MRI control group.

Procedure

Instructions were first read aloud to explain that the task involved the recall of details about a specific recent event (within the last year) for each memory that was personally experienced at a particular time and place, and which occurred over minutes and hours but no longer than one day. The instructions also directed participants to generate as much detail as possible about the selected event in response to a series of retrieval cues. Participants were encouraged to free associate, if necessary, in order to facilitate the selection of an appropriate specific event. It was explained that the participant should feature in the autobiographical event and that the participant was free to choose any memory that was compatible with the instructions. Participants were then shown the written instructions. In line with the standardised administration, structured specific probes were provided after the general probes to target and facilitate the recollection of further contextual details from five discrete categories (event, time, place, perceptual, emotion/thought details). Participants were given a time limit of 5 min per event.

Scoring

All event descriptions were recorded on a digital recorder for subsequent transcription and scoring. The events were verified, where possible, by asking relatives and/or friends of the participants. Events were scored in accordance with the standardised procedure of the autobiographical interview, which involved the segmentation of each event into informational details (bits) that were classified as internal or external. Internal details reference the main episode being described, were situated within a specific spatiotemporal context, convey episodic re-

experiencing, and thereby correspond to a quantitative measure of episodic memory. Internal details were assigned to one of the five response categories (event, time, place, perceptual, emotion/thought details). By contrast, external details refer to details that were not related to the main event, metacognitive statements or editorialising, repetitions, or semantic facts. Internal and external details were cumulatively summated to derive internal (episodic) and external (semantic) composite scores (Figure 2B).

Two trained raters independently scored all of the events acquired from LGI1 VGKC-complex-Ab LE patient group and age-matched control participants. Intra-class correlation coefficients were calculated to assess inter-rater reliability.

Results

Neuroimaging

Hippocampal subfield segmentation: LGI1-antibody LE subgroup analysis

Re-analysis of the patient group hippocampal subfield segmentation data after removing the non-LGI1 patient (i.e., with n=17 LGI1-antibody positive patients) indicated that the results held both in terms of the main effects, interaction terms, and with the planned comparisons as compared to the results conducted with n=18. In particular, a three-way mixed-model ANOVA was conducted on the subfield volumes of the 17 LGI1-antibody patients, with two within-subjects variables (subfield and side) and one between-subjects variable (group). Mauchly's test demonstrated that the assumption of sphericity had been violated ($\chi^2_{(9)}$=81.16, p<0.0001), therefore, degrees of freedom were corrected using Greenhouse-Geisser estimates (ε=0.48). There were significant main effects of group ($F_{(1,32)}$=5.95, p=0.020), side ($F_{(1)}$=42.59, p<0.0001), and subfield ($F_{(1.83)}$= 308.54, p<0.0001). Significant two-way interactions were observed between group and subfield ($F_{(1.83)}$=3.99, p=0.027) and between side and subfield ($F_{(1.91,61.10)}$=19.07, p<0.0001). The three-way interaction between group, subfield and side was not significant ($F_{(1.91)}$=1.65, p=0.202).

Also in line with the results based on all 18 LGI1 VGKC-complex-Ab patients, the planned group comparisons revealed a significant reduction in total CA3 volume–again, collapsed across left and right CA3 due to the absence of a significant group interaction with side and subfield–of the LGI1-antibody LE patients (mean=375 (SD=84) relative to age-matched controls (mean=531 (SD=128)) ($F_{(1,32)}$=17.58, p=0.0001; Cohen's d=1.44), whereas the group

differences in subiculum, CA1, CA2, and dentate gyrus volumes were not statistically significant at the alpha criterion corrected for multiple comparisons (Cohen's *d* all <0.8).

Evidence of a restricted subfield lesion profile in both the LGI1-antibody LE subgroup and LGI1 VGKC-complex-Ab LE group is different from the generalised hippocampal atrophy seen in conditions such as Alzheimer's disease, where all subfields except CA2 are implicated (Wisse *et al.*, 2014b), and may help to explain the differences in memory pathology. More broadly, evidence of a loss of CA3 integrity suggests that computational mechanisms ascribed to CA3 are also likely to be impaired in the patients (Rolls, 2013; Deuker *et al.*, 2014), because disrupted binding of separate properties of an object, such as orientation and location, has been reported in patients with VGKC-complex antibodies (Pertzov *et al.*, 2013).

CA3 atrophy was unaffected by T2-weighted MRI signal hyperintensities on presentation

A three-way Kruskal-Wallis Test comparing CA3 subfield volume between patients with T2-weighted MRI signal hyperintensities on presentation (n=5), no signal change on presentation (n=13), and the age-matched controls revealed a significant between-group different ($\chi^2_{(2)}$=11.99, p=0.002). Post hoc tests revealed no significant difference in CA3 volume between the patients with and without T2-weighted MRI signal hyperintensities (Dunn's z-test-statistic =-1.72, p=0.756), whereas there were significant reductions in CA3 volume between the patients with T2-weighted signal hyperintensities on presentation and controls (Dunn's z-test-statistic=-13.36, p=0.012) and patients without T2-weighted signal hyperintensities and controls (Dunn's z-test-statistic=-11.63, p=0.002).

Additional analyses of CA3 atrophy

The nature of the CA3 atrophy was additionally assessed in two ways. First, individual patient CA3 volumes were compared against the control mean CA3 volume. This demonstrated a mean CA3 proportion of 0.72 (SEM=0.05) on the left and 0.72 (SEM=0.02) on the right. A relative reduction compared to the control mean CA3 volume was seen in 17/18 participants on the left and 18/18 on the right. As in the case of the planned group comparison reported earlier, an independent-samples t-test indicated that there was no significant between left and right CA3 atrophy ($t_{(17)}$=0.17, p=0.865). Second, we assessed whether the overall contribution of CA3 to the total hippocampal volume differed between left and right hippocampi. The proportions were

calculated by dividing the left and right CA3 volumes by the total left and right hippocampal volumes, respectively. This demonstrated that for the patients the proportion of the total hippocampus volume constituted by CA3 was 0.143 (SEM=0.005) on the left and 0.144 (SEM=0.005) on the right, with no significant difference between these values ($t_{(17)}$=-0.20, p=0.844).

CA3 atrophy and VGKC-complex antibody titre on presentation

High positive VGKC-complex-Ab titres (>400 pmol/l) are associated autoimmune limbic encephalitis that is responsive to immunotherapy (Paterson *et al.*, 2014), and with cognitive impairment and seizures (Klein *et al.*, 2013). By contrast, the relevance of lower antibody titres (≤400 pmol/l) for diagnosis or management appears equivocal (Paterson *et al.*, 2014), and may not differ when compared with high positive titres in terms of memory outcome (Malter *et al.*, 2014). Nonetheless, lower antibody titres may be relevant for clinical evaluation when they co-occur with cell surface antigens leucine-rich glioma inactivated 1, contactin associated protein 2 or both, which was found in 61% of patients when titres were ≤400 pmol/l (Klein *et al.*, 2013). As noted in the main text, the link between the antibody titre and CA3 pathology was tested by means of a linear regression with Huber correction and did not reveal evidence of a significant association (β_1=-0.002, R^2=0.07, t=1.07, p=0.30).

Table S1 Patient antibody, VGKC-complex-Ab concentration on presentation and CA3 *z*-score

Patient	Autoantibody subtype	VGKC-complex-Ab conc. on presentation (pmol/l)	CA3 *z*-score
001	LGI1	484	-1.24
002	LGI1	377	-.53
003	LGI1	461	-.84
003	LGI1	2099	-1.16
004	LGI1	5705	-1.03
005	LGI1	5409	-.70
006	LGI1	3181	-1.23
007	LGI1	1097	-1.49
008	LGI1	3856	-2.16
009	LGI1	3010	-1.27
0010	LGI1	4717	-2.38
0011	LGI1	4121	-1.53
0012	LGI1	1844	-.85
0013	LGI1	1798	.37
0014	LGI1	918	-.52

0015	LGI1	703	-1.34
0016	LGI1	205	-1.99
0017	LGI1	870	-.89
0018	VGKC-c	377	-1.24

Hippocampal CA3 subfield *z*-scores represent the combined (left and right hemisphere) total CA3 volume *z*-transformed relative to the age-matched control group. LGI1: leucine-rich glioma inactivated-1 antibody positive. VGKC-complex-Ab conc. on presentation: voltage-gated postassium channel-complex antibody concentration on presentation. VGKC-c: voltage-gated postassium channel-complex antibody positive.

Behaviour

Autobiographical Interview

Autobiographical interview data were acquired from 16 patients in the amnesic group and 16 participants in the age-matched control group. Inter-rater reliability for the full set of data scored by two raters – as assessed by conducting a two-way mixed model design for absolute agreement – indicated a high-level of consistency in scoring, as determined by a high intraclass correlation coefficient, 0.92.

As summarised in the main text, a two-way mixed-model ANOVA was conducted on the postmorbid autobiographical interview cumulative point scores, with one within-subjects variable (memory detail type: internal (episodic), external (semantic)) and one between-subjects variable (group: LGI1 VGKC-complex-Ab LE patients, age-matched controls). Mauchly's test demonstrated that the assumption of sphericity was not violated. There were significant main effects of group ($F_{(1,30)}=11.59$, $p=0.002$) and detail type ($F_{(1,30)}=123.24$, $p<0.0001$), and a significant interaction between group and memory detail type ($F_{(1,30)}=18.07$, $p<0.0001$). Planned group comparisons, conducted with an alpha criterion corrected for multiple comparisons with the Bonferroni-Holm method, revealed that there was a significant reduction in internal (episodic) ($F_{(1,30)}=14.94$, $p<0.001$, Cohen's $d=1.37$) but not external (semantic) ($F_{(1,30)}=0.71$, $p=0.41$, Cohen's $d=0.30$) details (See Figure 2B).

The selective impairment of internal (episodic) details is consistent with evidence from our studies describing the effects of hippocampal damage – characterised at 3.0-Tesla – in six of the 18 patients reported here (McCormick *et al.*, 2016, 2017) and with other reports of hippocampal and extra-hippocampal damage (Rosenbaum *et al.*, 2008; Race *et al.*, 2011), and is

associated with a similar level of internal detail generated to that associated with left temporal lobe epilepsy (mean=27.6 (SD=24.4)) (Addis *et al.*, 2007). Damage to the MTL that extends beyond the hippocampus or neocortical involvement impairs non-episodic retrieval, whereas more focal lesions preferentially impair episodic detail retrieval - a finding that appears to be consistent across 147 cases of hippocampal amnesia (Spiers *et al.*, 2001). Evidence of no significant impairment on the Graded Naming Test (n=17, ave. z-score=0.70, s.e.m.=0.25, t-score$_{(16)}$=3.07, p=0.003) indicates that the episodic autobiographical deficit was not related to reduced verbal output/fluency.

References

Addis DR, Moscovitch M, McAndrews MP. Consequences of hippocampal damage across the autobiographical memory network in left temporal lobe epilepsy. Brain 2007; 130(Pt 9): 2327-42.

Ashburner J. A fast diffeomorphic image registration algorithm. Neuroimage 2007; 38(1): 95-113.

Ashburner J, Friston KJ. Unified segmentation. Neuroimage 2005; 26(3): 839-51.

Baddeley AD, Emslie H, Nimmo-Smith I. Doors and people: a test of visual and verbal recall and recognition. Bury St. Edmunds, England: Thames Valley Test Company; 1994.

Bontempi B, Laurent-Demir C, Destrade C, Jaffard R. Time-dependent reorganization of brain circuitry underlying long-term memory storage. Nature 1999; 400(6745): 671-5.

Bozeat S, Lambon Ralph MA, Patterson K, Garrard P, Hodges JR. Non-verbal semantic impairment in semantic dementia. Neuropsychologia 2000; 38: 1207–15.

de Flores R, La Joie R, Landeau B, Perrotin A, Mezenge F, de La Sayette V*, et al.* Effects of age and Alzheimer's disease on hippocampal subfields: comparison between manual and FreeSurfer volumetry. Hum Brain Mapp 2015; 36(2): 463-74.

Delis DC, Kaplan E, Kramer J. Delis Kaplan Executive Function System. San Antonio, TX: The Psychological Corporation; 2001.

Deuker L, Doeller CF, Fell J, Axmacher N. Human neuroimaging studies on the hippocampal CA3 region - integrating evidence for pattern separation and completion. Front Cell Neurosci 2014; 8: 64.

Frankland PW, Bontempi B. The organisation of recent and remote memories. Nat Rev Neurosci 2005; 6: 119-30.

Frisch C, Malter MP, Elger CE, Helmstaedter C. Neuropsychological course of voltage-gated potassium channel and glutamic acid decarboxylase antibody related limbic encephalitis. Eur J Neurol 2013; 20(9): 1297-304.

Herranz-Perez V, Olucha-Bordonau FE, Morante-Redolat JM, Perez-Tur J. Regional distribution of the leucine-rich glioma inactivated (LGI) gene family transcripts in the adult mouse brain. Brain Res 2010; 1307: 177-94.

Hurley AC, Al-Radaideh A, Bai L, Aickelin U, Coxon R, Glover P, *et al.* Tailored RF pulse for magnetization inversion at ultrahigh field. Magn Reson Med 2010; 63(1): 51-8.

Keller SS, Roberts N. Voxel-based morphometry of temporal lobe epilepsy: an introduction and review of the literature. Epilepsia 2008; 49(5): 741-57.

Klein CJ, Lennon VA, Aston PA, McKeon A, O'Toole O, Quek A, *et al.* Insights from LGI1 and CASPR2 potassium channel complex autoantibody subtyping. JAMA neurology 2013; 70(2): 229-34.

Levine B, Svoboda E, Hay JF, Winocur G, Moscovitch M. Aging and autobiographical memory: dissociating episodic from semantic retrieval. Psychology and Aging 2002; 17: 677-89.

Lux V, Atucha E, Kitsukawa T, Sauvage MM. Imaging a memory trace over half a life-time in the medial temporal lobe reveals a time-limited role of CA3 neurons in retrieval. eLife 2016; 5.

Malone IB, Leung KK, Clegg S, Barnes J, Whitwell JL, Ashburner J, *et al.* Accurate automatic estimation of total intracranial volume: a nuisance variable with less nuisance. Neuroimage 2015; 104: 366-72.

Malter MP, Frisch C, Schoene-Bake JC, Helmstaedter C, Wandinger KP, Stoecker W, *et al.* Outcome of limbic encephalitis with VGKC-complex antibodies: relation to antigenic specificity. J Neurol 2014; 261(9): 1695-705.

McCormick C, Rosenthal CR, Miller TD, Maguire EA. Deciding what is possible and impossible following hippocampal damage in humans. Hippocampus 2017. 10.1002/hipo.22694

McCormick C, Rosenthal CR, Miller TD, Maguire EA. Hippocampal Damage Increases Deontological Responses during Moral Decision Making. J Neurosci 2016; 36(48): 12157-67.

McDermott KB, Szpunar KK, Christ SE. Laboratory-based and autobiographical retrieval tasks differ substantially in their neural substrates. Neuropsychologia 2009; 47(11): 2290-8.

Mougin O, Abdel-Fahim R, Dineen R, Pitiot A, Evangelou N, Gowland P. Imaging gray matter with concomitant null point imaging from the phase sensitive inversion recovery sequence. Magn Reson Med 2015.

Nadel L, Moscovitch M. Memory consolidation, retrograde amnesia and the hippocampal complex. Curr Opin Neurobiol 1997; 7(2): 217-27.

Osterrieth PA. Le test de copie d'une figure complexe: contribution à l'étude de la perception et de la mémoire [The Complex Figure Test: Contribution to the study of perception and memory]. Arch Psychol 1944; 28: 1021–34.

Palombo DJ, Alain C, Soderlund H, Khuu W, Levine B. Severely deficient autobiographical memory (SDAM) in healthy adults: A new mnemonic syndrome. Neuropsychologia 2015; 72: 105-18.

Paterson RW, Zandi MS, Armstrong R, Vincent A, Schott JM. Clinical relevance of positive voltage-gated potassium channel (VGKC)-complex antibodies: experience from a tertiary referral centre. J Neurol Neurosurg Psychiatry 2014; 85(6): 625-30.

Pertzov Y, Miller TD, Gorgoraptis N, Caine D, Schott JM, Butler C*, et al.* Binding deficits in memory following medial temporal lobe damage in patients with voltage-gated potassium channel complex antibody-associated limbic encephalitis. Brain 2013; 136(Pt 8): 2474-85.

Race E, Keane MM, Verfaellie M. Medial temporal lobe damage causes deficits in episodic memory and episodic future thinking not attributable to deficits in narrative construction. J Neurosci 2011; 31(28): 10262-9.

Robertson IH, Ward T, Ridgeway V, Nimmo-Smith I. The Test of Everyday Attention. Bury St. Edmunds, England: Thames Valley Test Company; 1994.

Roediger HL, 3rd, McDermott KB. Two types of event memory. Proc Natl Acad Sci USA 2013; 110(52): 20856-7.

Rolls ET. A quantitative theory of the functions of the hippocampal CA3 network in memory. Front Cell Neurosci 2013; 7: 98.

Rosenbaum RS, Moscovitch M, Foster JK, Schnyer DM, Gao F, Kovacevic N*, et al.* Patterns of autobiographical memory loss in medial-temporal lobe amnesic patients. J Cogn Neurosci 2008; 20(8): 1490-506.

Spiers HJ, Maguire EA, Burgess N. Hippocampal amnesia. Neurocase 2001; 7(5): 357-82.

Squire LR, Alvarez P. Retrograde amnesia and memory consolidation: a neurobiological perspective. Curr Opin Neurobiol 1995; 5(2): 169-77.

Wagner J, Weber B, Elger CE. Early and chronic gray matter volume changes in limbic encephalitis revealed by voxel-based morphometry. Epilepsia 2015; 56(5): 754-61.

Warrington EK. Recognition Memory Test: Manual. Berkshire, UK: NFER-Nelson; 1984.

Wechsler D. Wechsler Memory Scale. 3rd ed. New York: The Psychological Corporation; 1997.

Weschler D. Wechsler Adult Intelligence Scale. 3rd ed. San Antonio, TX: The Psychological Corporation; 1997.

Wisse LE, Biessels GJ, Geerlings MI. A critical appraisal of the hippocampal subfield segmentation package in FreeSurfer. Front Aging Neurosci 2014a; 6: 261.

Wisse LE, Biessels GJ, Heringa SM, Kuijf HJ, Koek DH, Luijten PR*, et al.* Hippocampal subfield volumes at 7T in early Alzheimer's disease and normal aging. Neurobiol Aging 2014b; 35(9): 2039-45.

Yushkevich PA, Piven J, Hazlett HC, Smith RG, Ho S, Gee JC*, et al.* User-guided 3D active contour segmentation of anatomical structures: significantly improved efficiency and reliability. Neuroimage 2006; 31(3): 1116-28.
